# Poly (*N*-isopropylacrylamide) Microgel-Based Optical Devices for Sensing and Biosensing

**DOI:** 10.3390/s140508984

**Published:** 2014-05-21

**Authors:** Molla R. Islam, Andrews Ahiabu, Xue Li, Michael J. Serpe

**Affiliations:** Department of Chemistry, University of Alberta, Edmonton, AB, T6G 2G2, Canada; E-Mails: molla1@ualberta.ca (M.R.I.); ahiabu@ualberta.ca (A.A.); xue13@ualberta.ca (X.L.)

**Keywords:** stimuli-responsive polymers, sensors and biosensors, photonic materials, poly (*N*-isopropylacrylamide)-based microgels, etalons

## Abstract

Responsive polymer-based materials have found numerous applications due to their ease of synthesis and the variety of stimuli that they can be made responsive to. In this review, we highlight the group's efforts utilizing thermoresponsive poly (*N*-isopropylacrylamide) (pNIPAm) microgel-based optical devices for various sensing and biosensing applications.

## 1. Introduction

Stimuli-responsive polymeric materials are capable of responding chemically and/or physically to changes in their environment. Ideally the response is reversible, *i.e.*, once the stimulus is withdrawn, the responsive polymer/material reverts back to its initial state. Among all responsive polymers and polymer-based systems, thermoresponsive poly (*N*-isopropylacrylamide) (pNIPAm) is the most well studied, and exhibits an abrupt conformational change at a temperature ≈ 32 °C [1–6]. Specifically, pNIPAm is fully water soluble, and swollen (extended conformation), at temperatures <32 °C and transitions to a hydrophobic globular conformation (collapsed) state at temperatures >32 °C [7,8]. PNIPAm can also be crosslinked to generate hydrogels and/or hydrogel particles (microgels). These materials also transition from a water solvated hydrophilic state, to a collapsed, hydrophobic state as their temperature is increased to >32 °C. This solvation state change makes them superb candidates for a number of different applications [9–16]. Additional functionality can easily be introduced into pNIPAm hydrogels/microgels by simply introducing a functional comonomer at the time of synthesis. The most common functional groups added to these materials are acids/bases such as: acrylic acid (AAc), methacrylic acid (MAc) and amine-containing N-(3-aminopropyl)methacrylamide hydro-chloride (APMAH) [1,10,11,17–26]. The introduction of these functional groups makes the materials further responsive to other stimuli, e.g., solution pH. Furthermore, the functional groups can serve as reactive “handles” for further reaction with other functional molecules.

**Figure 1.**
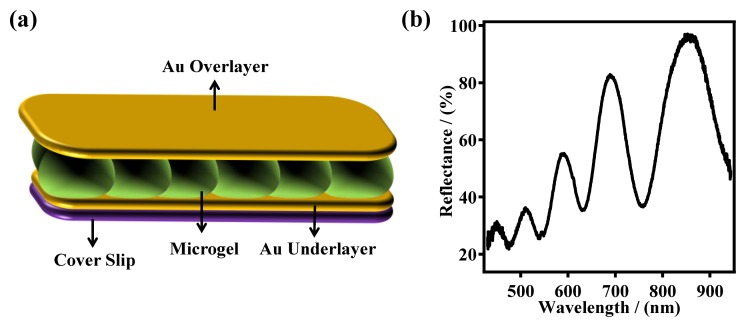
(**a**) The basic structure of a microgel-based etalon. The Au overlayer in the figure is drawn as a planar layer, but is actually conformal to the microgel layer. Each Au layer was supported by 2 nm Cr as an adhesion layer. (**b**) A representative reflectance spectrum from a poly(*N*-isopropylacrylamide)-co-acrylic acid, (pNIPAm-co-AAc) microgel-based etalon in pH 6.5 solution. Reproduced with permission from [12].

In our group, the most common functional group added to pNIPAm-based microgels is AAc, which has a pKa of ∼4.25. Therefore, the microgels exhibit negative charges at pH > 4.25 due to the deprotonation of AAc, and are neutral at pH < 4.25. Additionally, at high pH, the microgels exhibit a large diameter, relative to low pH, due to the Coulombic repulsion between the microgels negative charges. Our group has utilized pNIPAm-based microgels for a variety of applications, including sensing/biosensing, water remediation, controlled/triggered drug delivery, and as aicial muscles [11,22,27–34]. Many applications rely on the group's microgel-based etalon technology; an etalon is an optical device that we fabricate by sandwiching a monolithic monolayer of pNIPAm-based microgel particles between two thin Au layers. These devices exhibit visual color and characteristic multipeak reflectance spectra, as can be seen in Figure 1 [11,21,24,28,35–38]. The device structure is fabricated by “painting” a concentrated solution of pNIPAm-based microgels on a Cr/Au coated glass substrate [37]. The excess microgels not directly bound to the Au layer are washed away, and the layer dried followed by the deposition of another layer of Cr/Au on top of the microgel layer. The device structure is shown in Figure 1a. As mentioned above, this device exhibits multipeak reflectance spectra (Figure 1b); the position of the peaks in the reflectance spectra can be predicted from Equation (1):

(1) λm=2ndcosθ

where *n* is the refractive index of the dielectric layer, *d* is the mirror-mirror distance, *θ* is the angle of incident light relative to the normal, and *m* (an integer), is the order of the reflected peak.

## 2. Applications of Poly (*N*-isopropylacrylamide)-Based Microgels and Microgel-Based Etalons

### 2.1. Sensing Solution Temperature and pH

**Figure 2.**
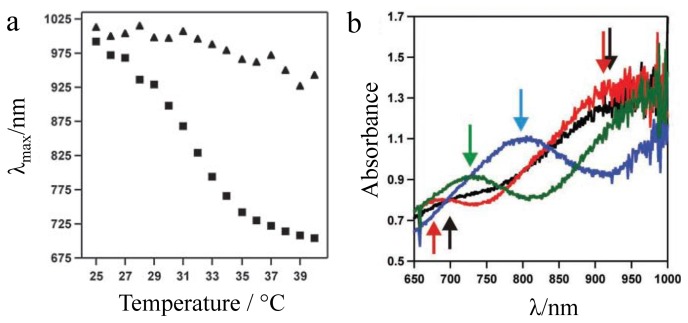
(**a**) *λ* max as a function of temperature for pNIPAm-co-AAc microgel-based etalons at (squares, ■) pH 3.0, and at (triangles, ▲) pH 6.5. (**b**) Absorbance (actually reflectance) as a function of λ at different temperatures; (black) 25 °C, (red) 29 °C, (blue) 33 °C, and (green) 37 °C. Reproduced with permission from [21].

Our early studies with pNIPAm microgel-based etalons were focused on characterizing their response to temperature. To characterize this, pNIPAm microgel-based devices were fabricated and their thermoresponsivity characterized in water. As shown in Figure 2, these devices are extremely sensitive to solution temperature. Specifically, the reflectance peak(s) shift ∼300 nm (blue shift) in response to a 15 °C change in the solution temperature [20]. We attributed the blue shift to the collapse of the microgels at high temperature, thus decreasing the distance between the etalons Au layers, as can be predicted from Equation (1). This was confirmed by measuring the thickness of the etalons at low and high temperature using atomic force microscopy (AFM) [20].

**Figure 3.**
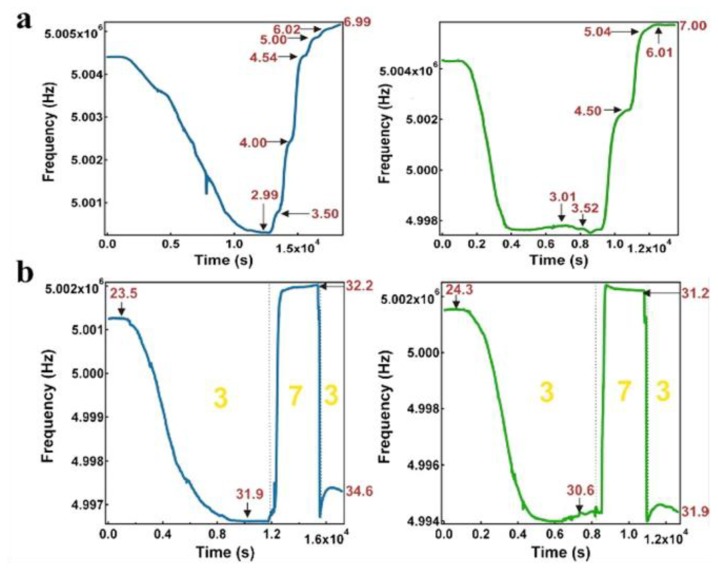
(**a**) The pH response of a pNIPAm-co-AAc microgel coated QCM crystal (left) and the same substrate after deposition of a 2 nm Cr/15 nm Au overlayer (right). In each case, the films were exposed to pH 3.0 water and the temperature of the solution adjusted to the film's lower critical solution temperature of ∼31–32 °C. Following stabilization of the QCM resonant frequency, the pH of the solution was adjusted stepwise by addition of 1 M NaOH. The actual pH values are indicated on each plot. (**b**) The pH response of a pNIPAm-co-AAc microgel coated QCM crystal (left) and the same substrate after deposition of a 2 nm Cr/15 nm Au overlayer (right). Initially, each device was heated to the film's lower critical solution temperature in pH 3.0 water. Following stabilization, the pH of the water was increased to 7.0 by addition of 1 M NaOH. Again, the signal was allowed to stabilize, and the pH returned to 3.0 by addition of concentrated HCl. The pH and temperature (°C) values are indicated on the individual plots. Reproduced with permission from [20].

Our group has also extensively studied pH responsive microgels, and microgel-based etalons. For example, pNIPAm-co-acrylic acid (pNIPAm-*co*-AAc) microgels have been synthesized and etalons subsequently constructed. While we have shown that the optical properties of the etalons depend on pH, we have also shown that etalons deposited on the surface of a quartz crystal microbalance (QCM) can be used for very sensitive solution pH detection [19,39]. Specifically, it is well known that the resonant frequency of a QCM crystal is dependent on the viscosity of the solution it is contacting [28]. It is also known that collapsed microgels have a higher viscosity that swollen microgels [40]; this is also true for microgels in etalons. As stated above, AAc-modified microgels at high pH are swollen (low viscosity) at pH > 4.25, while they are relatively collapsed (high viscosity) at pH < 4.25. By monitoring the QCM resonant frequency, we were able to measure solution pH with very high sensitivity. Specifically, we first showed that the QCM resonant frequency consistently decreased as the solution (pH 3.0) temperature was increased, as shown in Figure 3. This is due to the systematic increase in the microgel's viscosity at they collapse at high temperature. When the solution temperature was ∼32 °C, the microgels were nearly completely collapsed, and the QCM resonant frequency was a minimum. At this temperature, the solution pH was subsequently increased pH 7.0, which caused the microgels to swell. The swelling yielded a dramatic decrease in solution viscosity, and a concomitant increase in the QCM resonant frequency. By comparison of the panels in Figure 3, one can see that the etalons were more sensitive to solution pH changes that QCM crystals coated with just a microgel layer. We attributed this to the Au layer mass, initially near the QCM surface at pH 3.0, allowing for a greater frequency change when the microgels are swollen, and the Au is moved away from the QCM surface, when the solution is increased to pH 7.0. Therefore, the QCM response is a combination of both the viscosity decrease from the microgel swelling, and a mass decrease from the Au moving away from the QCM crystal. We showed that the response to pH was fully reversible over many cycles, as can be seen in Figure 3. Finally, we showed that the magnitude of the response, and hence sensitivity, depended on the thickness of the Au layer used to coat the microgels [27]. This phenomenon has been attributed to the increase in the mass of the Au on the microgels.

**Figure 4.**
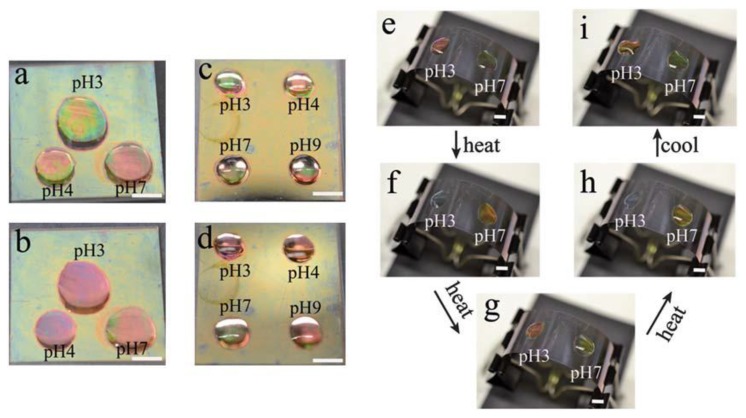
Photographs of an etalon with solutions of various pH's spotted on a single surface at (**a**,**c**,**e**,**i**) 25 °C and (**b**,**d**,**f**,**g**,**h**) 37 °C. (**f**) 3 min after heating; (**g**) 5 min after heating; (**h**) 6 min after heating. In each panel, the scale bar is 5 mm. Reproduced with permission from [37].

In another study, we showed that spatially isolated regions of a single microgel-based etalon were capable of exhibiting independent responses to solution pH and temperature [36]. In this investigation, we fabricated pNIPAm-co-AAc etalons, and independent drops of solutions (pH 3.0, 4.0, and 7.0) were added to the device surface. Three separate reflectance probes were added to the drops, and the etalons response to pH in those separate regions characterized. We observed that each reflectance spectrum behaved in a completely independent manner. Furthermore, the etalon's response can be visually observed, as can be seen in Figure 4. This observation could be used for a number of sensing applications as well as for display technologies.

### 2.2. Ion Sensing

**Figure 5.**
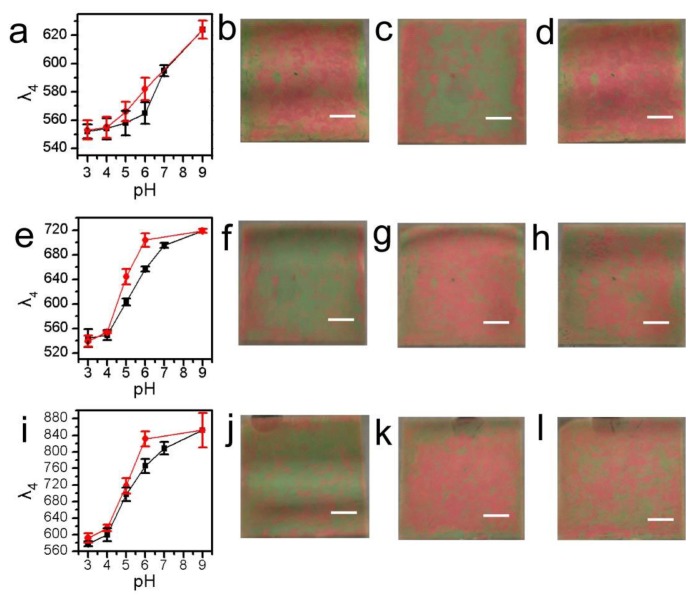
*λ*_4_ (here 4 represents the order of the reflectance peak defined in Equation (1)) of an etalon at 25 °C as a function of pH increasing (black curve) and pH decreasing (red curve) with I.S. (**a**–**d**) 0, (**e**–**h**) 2 and (**i**–**l**) 10 mM adjusted with NaCl. The average λ_4_ from three spectra obtained from different regions of the same etalon is shown with the error bars indicating ± one standard deviation. Photographs of the etalon in (b,f,j) pH 6.0, (c,g,k) pH 9.0, and (d,h,l) pH 6.0 (decreasing from pH 9.0) solution. The scale bars in the photographs are 0.5 cm. Reproduced with permission from [42].

In a recent study, pNIPAm-co-AAc microgel-based etalons were fabricated, and their response to solution pH in the presence of various salts characterized [41]. Specifically, an etalon was immersed in a solution containing different salts (K^+^, Na^+^ and NH_4_^+^) and the solution pH increased and decreased; the hysteresis of the device response was then characterized, and it was determined to depend dramatically on the elemental composition of the salt. The results are shown in Figure 5, which reveals that solutions composed of NH_4_^+^ exhibit the strongest hysteresis, followed by K^+^, and finally Na^+^. This observation was explained by the ion-pairing theory; such that if the water affinities for cations are closer to that of carboxylic acid groups, they exhibit stronger binding force. Not only does the device respond to different cations but also different concentrations of the same cation. For instance, increasing the concentration of NaCl, resulted in a significant increase in the hysteresis observed (Figure 5).

### 2.3. Sensing Biomolecules

**Figure 6.**
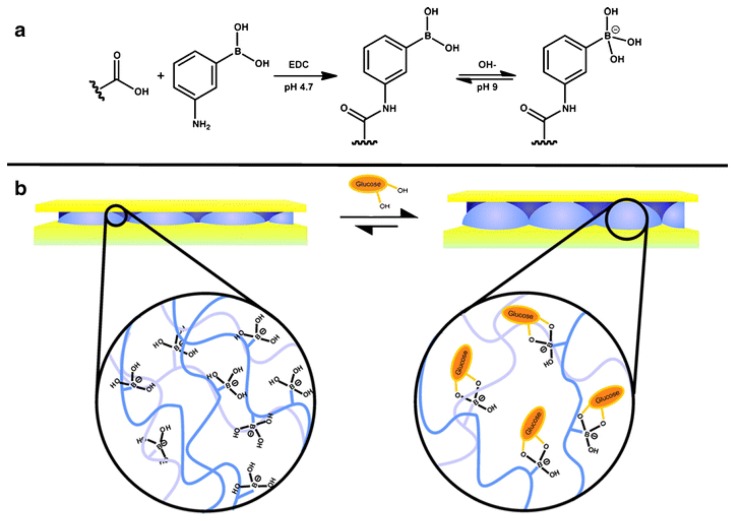
Reaction scheme for (**a**) the functionalization of the acrylic acid moieties on the microgel with 3-aminophenylboronic acid (APBA) followed by the activation of the boronic acid with base and (**b**) a cartoon depiction of the glucose responsivity of an APBA-functionalized microgel etalon at pH 9. Reproduced with permission from [23].

PNIPAm microgel-based technologies have been used previously by various groups for sensing and biosensing [1,10,18,42–48]. Specifically, Hoare and Pelton developed novel microgels for sensing glucose [42] in solution by observing a change in the microgel diameter that occurred due to the glucose-microgel interaction. Utilizing similar microgels, we were able to detect glucose in solution using our etalon technology [49]. To accomplish this, we first painted pNIPAm-co-AAc microgels onto a Au-coated glass substrate, followed by modification of the microgels with 3-amino phenylboronic acid, (APBA) by coupling it to the microgel AAc groups via 1-ethyl-3-(3-dimethylaminopropyl) carbodiimide (EDC). Following this, another Au layer was deposited on the microgel layer to yield the etalon. The fabricated etalon was immersed into a basic buffer where the boronic acid moieties on the APBA (p*K*_a_ = 8.2) were hydroxylated such that the boron possessed a negative charge (Figure 6a). At this pH, the phenyl boronic acid moieties exist in equilibrium between the charged and uncharged state. But, the charged state is capable of binding diols, like glucose [42]. As glucose binds, more boronic acid groups must convert to the charged state in order to maintain the equilibrium, which effective lowers the APBA p*K*_a_. When glucose is present, the bound state is preferred leading more hydroxylation of the boron atoms into a charged form. This leads to an increase in the Coulombic repulsion of negative charges inside the microgel, thereby swelling the microgel (Figure 6b). As the microgel swells, the distance between two Au mirrors increases and we see a red shift in the reflectance spectra of the etalon, according to Equation (1).

**Figure 7.**
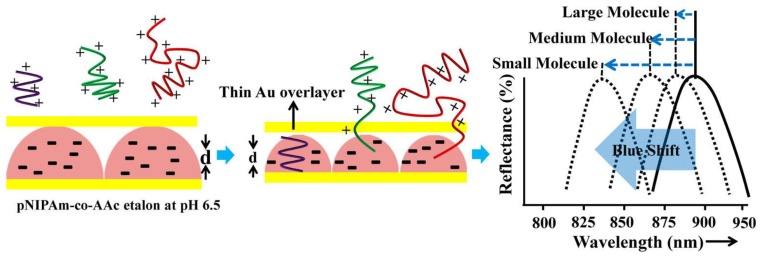
Polyelectrolyte penetration through the porous Au overlayer of an etalon. Reproduced with permission from [25].

In more recent publications [11,28], we showed that microgel-based etalons could be used to detect the concentration of streptavidin in solution. This approach was a result of a previous study, where we established that pNIPAm-co-AAc microgel-based etalons at high pH (*i.e.*, above the p*K*_a_ of AAc is ∼4.25) could deswell when exposed to poly(diallyldimethylammonium chloride) (pDADMAc), which is a positively charged linear polymer (polycation) [24]. This is a result of the pDADMAc penetrating the outer Au layer of the etalon, resulting in electrostatic interaction induced intra and intermicrogel crosslinking and collapse. This collapse led to an observable shift in the peaks of the reflectance spectrum as predicted from Equation (1). We found that the extent of the shift in peak position depended on the molecular weight (MW) and concentration of polycations as shown in Figure 7 [24]. We went on to show that this approach could be made sensitive to various polyelectrolyte molecular weights in solution, which could be used for characterizing polymers in a complex mixture [38]. For all experiments, the sides of the etalon were sealed with epoxy to ensure that the polycation only entered the etalon through the Au overlayer.

**Figure 8.**
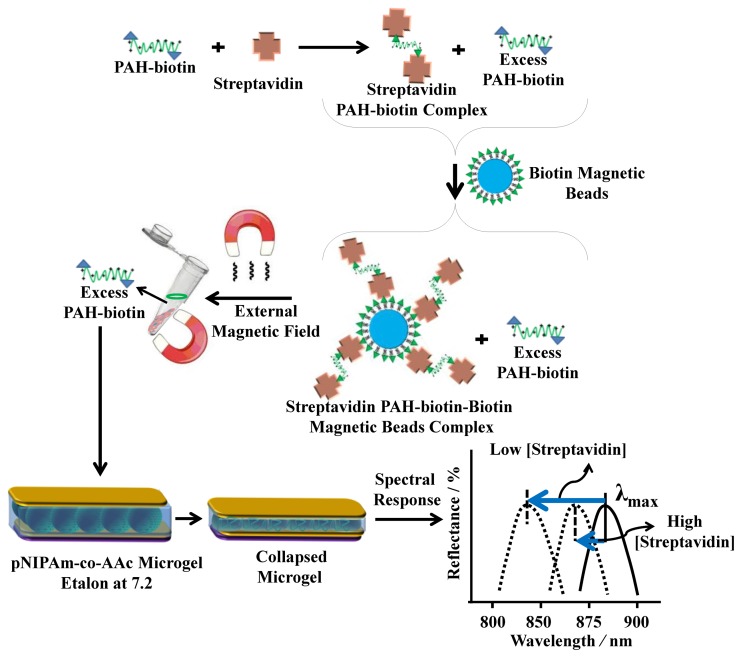
Streptavidin (the analyte) was added to an excess amount of biotin-modified poly (allylamine hydrochloride) (PAH). The PAH-biotin-streptavidin complex could then be removed from solution using biotin modified magnetic particles, leaving behind free, unbound PAH. The unbound PAH was subsequently added to a pNIPAm-co-AAc microgel-based etalon immersed in aqueous solution at a pH that renders both the microgel layer and the PAH charged. As a result, the etalon's spectral peaks shift in proportion to the amount of PAH-biotin that was added. This, in turn can be related back to the original amount of streptavidin added to the PAH-biotin. Reproduced with permission from [12].

For sensing streptavidin, we exploited the above phenomenon using the polycation poly(allylamine hydrochloride) (PAH) [11,28]. To accomplish this, PAH, which is “completely” charged at pH < ∼9.0, was modified with biotin (PAH-biotin). We showed that the PAH-biotin could also penetrate the etalon and crosslink the microgel layer leading to a spectral shift. Likewise, we found that the extent of the reflectance peak shift again depended on the amount of PAH-biotin added to the etalon until the etalon was “saturated” [11]. For sensing, we exposed aqueous solution of PAH-biotin to specific amounts of streptavidin; the concentration of PAH-biotin was always high enough to leave excess PAH-biotin in solution after all the PAH-biotin-streptavidin complexes have formed. Then, biotin modified magnetic particles were added to the solution, which bound to the PAH-biotin-streptavidin complexes. An external magnet was used to remove the magnetic particles bound with PAH-biotin-streptavidin from the solution. The solution containing the excess, unbound PAH-biotin was subsequently separated and added to the pNIPAm-co-AAc etalon stabilized in pH 7.2 (microgels negatively charged) solution maintained at 25 °C. When the PAH-biotin was added to the etalon, it resulted in a blue shift of the etalon's reflectance peaks (the sensing mechanism is shown in Figure 8). We found that the extent of the blue shift depends on the amount of streptavidin initially added to the PAH-biotin. Here, a low concentration of streptavidin initially present in solution yields a large amount of excess, unbound PAH-biotin after the reaction. When the excess PAH-biotin is added to the etalon, a large shift in the reflectance peaks was observed. Alternatively, a high concentration of streptavidin initially present in solution yields a small amount of excess, unbound PAH-biotin that is added to the etalon, which gives a small etalon response. It is important to note that we were able to get increasingly large spectral responses with decreasing analyte concentration.

## 3. Conclusions

Fabrication of pNIPAm-based optical devices could be achieved by sandwiching them between two thin Au layers. These devices (etalons) have been developed in the group for a variety of applications. In this submission, their use for detecting solution temperature, pH, ions, glucose, and streptavidin was detailed. We have also been able to expand on this technology for detection of specific DNA sequences in solution with the ability to differentiate a DNA sequence from another DNA sequence with only two base pair mismatches. This technology offers great promise as point-of-care diagnostics due to the ease of use, sensitivity, and the fact that each device cost ∼0.04 CAD. Therefore, their implementation in the developing world could potentially be achieved.
